# The essential impact of stress appraisals on work engagement

**DOI:** 10.1371/journal.pone.0291676

**Published:** 2023-10-18

**Authors:** Raghid Al Hajj, John G. Vongas, Muhammad Jamal, Ahmed R. ElMelegy

**Affiliations:** 1 Business Administration Department, Gulf University for Science and Technology, Mubarak Al-Abdullah, Kuwait; 2 Department of Management, Ithaca College School of Business, Ithaca, New York, United States of America; 3 Department of Management, John Molson School of Business, Concordia University, Montreal, Canada; Shenzhen University, CHINA

## Abstract

This paper explains the contradictory findings on the relationship between stress and work engagement by including appraisals as a driving mechanism through which job stressors influence engagement. In doing so, it explores whether stressors categorised as either challenging or hindering can be appraised simultaneously as both. Second, it investigates whether stress mindset explains not only how stressors are appraised, but also how appraisals influence engagement. Over five workdays, 487 Canadian and American full-time employees indicated their stress mindset and appraised numerous challenging and hindering stressors, after which they self-reported their engagement at work. Results showed that employees rarely appraised stress as uniquely challenging or hindering. Moreover, when employees harbored positive views about stress, stressors overall were evaluated as less hindering and hindrance stressors were particularly more challenging. Stress mindset appears to be critical in modulating the genesis of stress appraisals. In turn, appraisals explained the stressor-engagement relationship, with challenge and hindrance stressors boosting and hampering engagement, respectively. Finally, positive stress mindset buffered the negative effect of hindrance appraisals on engagement. Our findings clarify misconceptions about how workplace stressors impact engagement and offer novel evidence that stress mindset is a key factor in stress at work.

## 1 Introduction

Employee work engagement has been declining globally in recent years, and the COVID-19 pandemic promises to exacerbate its continued deterioration [1]. The job demands-resources model contends that job demands exhaust employees, both physically and mentally, while various job resources promote engagement [2, 3]. This central assumption of the model, however, overlooks the interpretive or ’appraised’ nature of employee stress that derives from those demands.

This paper addresses this oversight by shedding light on how employees perceive stress from daily job demands and how those perceptions influence their engagement. Scholars in occupational health psychology generally define job stressors as work conditions and events that bring about a slew of negative psychological (e.g., affective and cognitive states) and physical (e.g., elevated cortisol levels, psychosomatic complaints) outcomes affecting employees, outcomes that are collectively referred to as *strain* [4]. In addition to undermining the health and wellbeing of employees, these prevailing conditions range from mild chronic stressors to intense acute stressors [4]. Job stressors are prevailing conditions that undermine employee wellbeing and range from mild chronic stressors to intense acute stressors [5]. Whereas chronic work stressors accrue over time and have a high frequency of occurrence (e.g., overload, role conflict), acute stressors are momentary, potent, and less frequent (e.g., airline crew emergency landings) [6]. In most organisational settings, however, critical alterations occur less frequently. Also, research on stress has been largely driven by frameworks that involve the effects of day-to-day contexts on organisational behaviour rather than by events triggering acute stress [7]. With this in mind, our paper centers on chronic stress in organisations.

Cavanaugh and her colleagues [8] conceptualised stressors by drawing on Lazarus’s [9] transactional theory of stress, which posits that at the crux of every stress episode lies an individual’s *appraisal* of the event. Then, appraisals influence one’s emotions and behaviours. Thus, they constitute an important mechanism by which stress-inducing conditions outside the self can translate into outcomes manifested within the self. Cavanaugh et al. [8] defined challenge stressors as impositions that offer a person with growth opportunities (e.g., overload, work complexity). Hindrance stressors, however, are demands that thwart a person’s goal-seeking behaviour (e.g., role conflict and ambiguity) [10]. The literature proposes that these two types of stressors impact employee work engagement in distinct ways [11, 12], but has often taken for granted that individuals categorise stressors solely as either challenging or hindering. Moreover, people’s notion about the function of stress itself varies. Whereas some believe that stress improves their motivation, others are convinced that it is counterproductive.

Our objective is to understand how work stressors are appraised and, consequently, how these appraisals influence work engagement. We, therefore, investigate the appropriateness of categorising stressors strictly as either challenges or hindrances. Finally, because each person views the nature of stress through their own lens, we also account for a critical yet neglected boundary condition, namely stress mindset, which is the degree to which a person believes that stress has either enhancing or debilitating effects on stress-related job outcomes (e.g., performance, engagement, and wellbeing to name a few) [13]. While stress appraisals are related to a specific stressor, stress mindset is an overarching belief about the nature of stress itself, regardless of the source and its overall effects.

Two principal contributions emanate from this research. First, we attempt to shed light on how and why stress appraisals might be better suited to explain the variance in work outcomes than the challenge-hindrance stressor model. Mazzola and Disselhorst’s [14] recent review shows that research involving appraisals is grossly lacking and that their inclusion should help managers design better stress interventions. And second, we provide an account for the role of individual differences in stress mindset as this will enhance our understanding of how employee attitudes toward stress and its ramifications influence the relationships between stressors and stress appraisals, as well as between these appraisals and employee engagement. This knowledge promises to empower both managers and employees about optimal employee-job fit.

## 2 Theoretical background and conceptual development

### 2.1 In praise of stress appraisals

Dichotomizing stressors unilaterally as challenging or hindering originates from the stimulus-response model of stress and the assumption that reactions to stressors are homogeneous across individuals [15]. Challenge stressors are under one’s voluntary control and offer personal growth, while hindrance stressors are less controllable and obstruct one’s goal pursuits. This binary sorting violates the transactional theory of stress, which holds that individual and situational characteristics interact in a stress encounter to influence how much a person appraises the encounter to be *simultaneously* challenging and hindering [16]. As such, Mazzola and Disselhorst [14] quipped that the original challenge-hindrance model "fails to account for this appraisal process" (p. 951). Appraisals are thus the mechanism by which the stressor leads to immediate or proximal effects (e.g., emotions) as well as consequential or distal effects (e.g., social functioning). Disregarding challenge or hindrance appraisals and assuming that a particular stressor leads to pure challenge or pure hindrance appraisals is theoretically problematic and can lead to internally invalid conclusions as a portion of the variance in the appraisals of those stressors is disregarded.

Although limited to a subset of stressors, Webster et al. [17] found evidence of this mechanism in a study in which two challenge stressors (workload, responsibility) and two hindrance stressors (role conflict, ambiguity) were appraised equally as challenging and hindering. In particular, they demonstrated that correlations between a stressor and each of its appraisals could be similar, such as those between workload and challenge and hindrance appraisals (.29 and .23, respectively). Later, Gerich [18] came to a similar conclusion regarding appraisal equivalence, thus suggesting that stressors are not fixed binaries when it comes to how they are appraised but could be appraised as being both challenging and hindering. More recently, Li and colleagues showed that time urgency, a challenge stressor as identified by Cavanaugh et al. [8] demonstrated significant correlations with its challenge and hindrance appraisals in three studies published in two papers [19, 20]. The authors found a significant correlation between role conflict, a hindrance stressor as identified by Cavanaugh et al. [8], and both of its appraisals simultaneously in one study [19] and with challenge appraisals and hindrance appraisals in studies one and two, respectively, in another work [20]. A major reason why individuals evaluate stressors as both challenging and hindering is that both forms deplete one’s energy during coping, resulting in strain [21]. Even if the benefits of challenging stress offset the negative impact of strain on work outcomes, both appraisal types deplete one’s resources and require the person to resort to coping [11]. Hence and even though some stressors have been clustered as challenge stressors and others as hindrance stressors, both stressor types will still lead to challenge and hindrance appraisals simultaneously. Therefore, we hypothesize the following:

*Hypothesis 1*: *Challenge stressors are positively related to challenge appraisals*.
*Hypothesis 2*: *Challenge stressors are positively related to hindrance appraisals*.
*Hypothesis 3*: *Hindrance stressors are positively related to challenge appraisals*.
*Hypothesis 4*: *Hindrance stressors are positively related to hindrance appraisals*.

### 2.2 Stress mindset: Judging the utility of stress

Research exploring boundary conditions for the stressor-appraisal link is nascent. An exception is the work of LePine et al. [22], which looked at how leader charisma moderates employee appraisals of various challenge and hindrance stressors. Given that individual differences are a fixture in the transactional theory of stress, this exception seems odd. One critical and overlooked individual difference related to occupational stress is stress mindset, defined as "the extent to which one holds the belief that stress has enhancing consequences […] or holds the belief that stress has debilitating consequences" [13: p.716]. Unlike appraisals which evaluate the valence and intensity of stress felt in a specific time and context, stress mindset is a meta-cognitive belief about the nature and outcomes of stress [23]. Stress mindset is also distinct from seemingly similar personality dimensions, such as positive and negative affectivity, in that while these dimensions are distinct and orthogonal [24], positive and negative stress mindsets are the opposing boundaries of a continuum of beliefs about stress and its outcomes. In addition, and unlike the various personality traits that have been shown to have an influence on experiencing stress, stress mindset is more malleable and can be influenced via simple interventions such as an informative short video presentation (see the work of Crum et al. [13]). However, as a construct, stress mindset has been scarcely used to study challenge and hindrance stressors and their associated appraisals. This, in spite of its reputation of having a "significant impact on the manner in which stress is behaviorally approached as well as the manner in which stress is psychologically experienced [13: p.718]. With appraisals being psychological evaluations of stressors, we can see how mindset should be investigated as a critical boundary condition in the stressor-to-appraisal relation.

Compared to individuals with a negative stress mindset, those having a positive one interpret stress as more challenging and show improved affectivity and cognitive ability. They are more motivated to approach a stressor since they believe in its enhancing properties. As a result, they are also more likely to exhibit moderate increases in cortisol levels and approach-oriented behaviours when faced with stressors [21]. Perceptual selectivity, elevated optimism, and an affinity toward positive affect should make these individuals more likely to anticipate challenge-inducing characteristics in a certain stressor, regardless of its type, when they are present. On the other hand, individuals having a negative stress mindset are more likely to show greater cortisol fluctuations and are more likely to face stressors with an avoidance-oriented approach and negative affect and pessimism [21]. These individuals are more likely to obsess about the minutiae of the uncontrollable and hindering characteristics of the encountered stressor, again irrespective of how it is categorised in the literature. Accordingly, we propose that:

*Hypothesis 5*: *Stress mindset will moderate the positive relationship between challenge stressors and challenge appraisals; for individuals with a more positive mindset*, *the relationship will be stronger than for individuals with a less positive mindset*.
*Hypothesis 6*: *Stress mindset will moderate the positive relationship between challenge stressors and hindrance appraisals; for individuals with a more positive mindset*, *the relationship will be weaker than for individuals with a less positive mindset*.
*Hypothesis 7*: *Stress mindset will moderate the positive relationship between hindrance stressors and challenge appraisals; for individuals with a more positive mindset*, *the relationship will be stronger than for individuals with a less positive mindset*.
*Hypothesis 8*: *Stress mindset will moderate the positive relationship between hindrance stressors and hindrance appraisals; for individuals with a more positive mindset*, *the relationship will be weaker than for individuals with a less positive mindset*.

### 2.3 Stress and work engagement: Incorporating appraisals

Maslach and Leiter [25] conceptualised engagement as the opposite of burnout. Schaufeli et al. [26] considered it to be an independent concept, albeit negatively related to burnout, and defined it as a "work-related state of mind that is characterised by vigor, dedication, and absorption" (p.74). Vigor represents high energy, grit, and the desire to invest effort on a task. Dedication is feeling pride when faced with work challenges and absorption is being completely immersed in one’s work to the point that time passes unnoticeably. Thus, the two main points separating engagement from burnout is energy and an identification with one’s work. As such, organisations with engaged employees have higher shareholder returns and lower turnover and absenteeism [11].

In studies on engagement, researchers have been guided by the job demands-resources model [27], which holds that demands lead to burnout via a process that depletes one’s energy in contrast to resources that promote engagement by triggering motivation. Despite the model’s ability to predict burnout from demands, evidence for the effects of demands on engagement over the years has been ambiguous. As a result, scholars have concluded that demands alone—without considering *types* of demands—are problematic for predicting engagement. Crawford et al. [11] argued that such inconsistencies could be resolved if demands were differentiated into challenges/hindrances and found that the meta-analytic correlations between undifferentiated job demands and engagement improved significantly after dichotomisation. They also contended that few studies exist that measure stress appraisals as a means of predicting work-related outcomes [14]. However, neither Crawford et al. [11] nor Mazzola & Disselhorst [14] included appraisals in their meta-analyses.

Stressors evaluated as challenges improve thriving, positive affect, and approach motivation, in addition to cognitive performance and flexibility [23]. Challenges, being more controllable than hindrances, also facilitate goal pursuit by offering opportunities to satisfy achievement needs and contribute to health and wellbeing. Since engagement is a "persistent and pervasive affective-cognitive state" [26: p.74)], the challenge-inducing benefits of positive affectivity and cognitive performance and flexibility should reflect increased engagement. Hindrance appraisals represent hostile constraints on employees who, in turn, rely on emotion-based coping (e.g., withdrawal) [11]. They also frustrate basic needs like competence, autonomy, and relatedness and, consequently, impede goal achievement [28]. Moreover, they are negatively associated with wellbeing and are linked to cognitive deterioration and negative affect [23]. Employees appraising stressors as hindrances are less likely to invest in their job [27], which ought them to be less engaged. Thus, we posit that:

*Hypothesis 9*: *Challenge appraisals are positively related to work engagement*.
*Hypothesis 10*: *Hindrance appraisals are negatively related to work engagement*.

### 2.4 The role of stress mindset in the appraisal-engagement relationship

Of the studies exploring the differential effects of challenge and hindrance appraisals on work engagement, few have addressed individual differences (with one exception, see [29]). We believe that individuals with varying stress mindsets should experience engagement differently following a stressful period due in part to their accrual of personal resources. Defined as "psychological characteristics of the self that are associated with resiliency and that refer to the ability to control and impact one’s environment"," personal resources are integrated within leading stress theories [30: p.49]. Such resources include optimism, self-efficacy, and self-esteem, among others. Stress mindset is akin to a personal resource in the pantheon of dispositional constructs related to the study of stress. Individuals having a stress-is-enhancing mindset are more optimistic when stressed and more likely to perceive opportunities for achievement and control compared with those having a stress-is-debilitating mindset. As a personal resource, a positive stress mindset should lead employees not only to be able to deal better with job demands but also to have more access to job resources [31] compared to their negative stress mindset counterparts whose views of stress can be seen as creating more demands on their personal resources. The boosting hypothesis [2] claims that job resources are especially relevant under stressful job conditions and describes that high demands coupled with high resources should jointly predict a surge in engagement whenever individuals appraising the stress as challenging have a more positive than negative stress mindset.

Contrary to challenge appraisals, hindrance appraisals signal that a given situation is unmanageable and that opportunities for growth are lacking. The less a given stressor is evaluated as a hindrance, the more likely that employees will become engaged. Unlike people whose positive stress mindsets enable them to perceive a stressor’s rewarding features, those with negative stress mindsets ought to also experience less engagement because they ruminate about aspects of the hindrance stressor that block goal attainment [22]. Individuals with a negative stress mindset are more sensitive to the lack of control and the low probability of achievement associated with hindrance appraisals hence reducing their vigor, dedication, and absorption. The stress-is-debilitating mindset has also been associated with lower cognitive ability and flexibility and increased negative affect [23], all of which should make the adverse effect of hindrance appraisals on work engagement more pronounced. Again, by viewing stress mindset as a personal resource, one may be guided by the buffering hypothesis of the job demands-resources model—the opposite counterpart to the boosting hypothesis—which suggests that resources lessen the negative effects that high demands have on engagement [30]. A positive stress mindset should thus be valuable in mitigating the predicted negative effect of hindrance appraisals on work engagement.

*Hypothesis 11*: *Stress mindset will strengthen the positive relationship between challenge appraisals and work engagement; individuals with a more positive stress mindset will experience a higher level of work engagement than those with a less positive stress mindset*.
*Hypothesis 12*: *Stress mindset will weaken the negative relationship between hindrance appraisals and work engagement; individuals with a more positive stress mindset will experience a higher level of work engagement than those with a less positive stress mindset*.

As we elaborated before, the transactional theory of stress on which the challenge-hindrance model of stress was based, clearly indicates the role of appraisals as the mechanism by which the stressor leads to proximal and distal effects. Recently, a moderating role for appraisals in the stressor-outcome relationship has been proposed and tested in two cross-sectional studies. Li et al. [19, 20] in-depth exploration of the current literature shows that only a few studies have measured appraisals and tested them as a mechanism or conduit through which stressors affect business-related outcomes. Scholars investigating such a mechanism have found that when appraisals are taken into consideration, the effect of the stressor on the outcome is greatly diminished and becomes nonsignificant at times. For example, Gerich [18] showed that, across many work conditions, the relationship between various work stressors and health outcomes became nonsignificant after introducing challenge and hindrance appraisals into the model [32, 33]. Collectively, these findings propose that a good portion of the variance in stressors (influencing a myriad of outcomes) is carried through appraisals. Appraisals thus appear to be a key mediator in the stress-to-outcome link.

*Hypothesis 13*: *Challenge appraisals will mediate the relationship between challenge stressors and work engagement*.
*Hypothesis 14*: *Challenge appraisals will mediate the relationship between hindrance stressors and work engagement*.
*Hypothesis 15*: *Hindrance appraisals will mediate the relationship between hindrance stressors and work engagement*.
*Hypothesis 16*: *Hindrance appraisals will mediate the relationship between challenge stressors and work engagement*.

**Fig 1:**
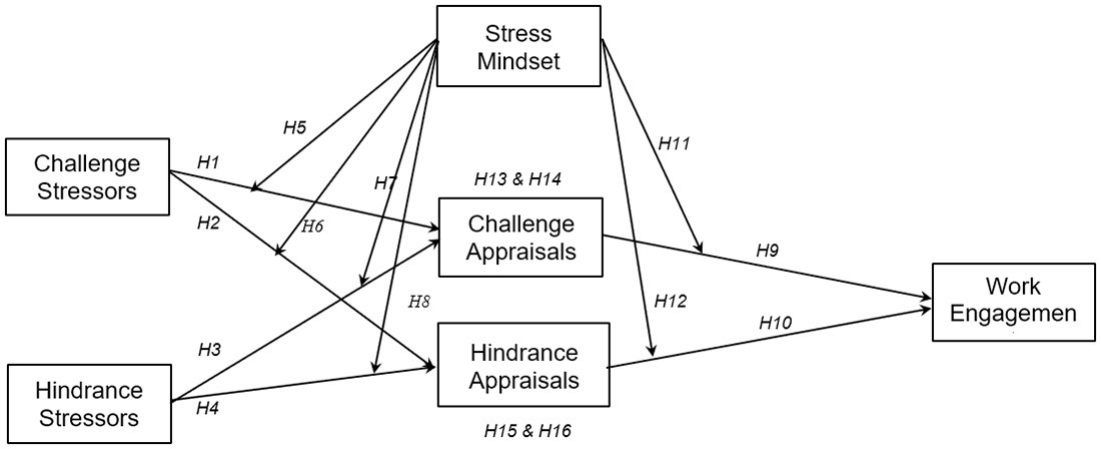
Conceptual model featuring tested hypotheses.

The hypothesis and our conceptual model are graphically represented in Fig 1.

## 3 Methods

### 3.1 Sample and procedure

Data was collected from incentivised participants and from volunteers to increase generalisability compared to most studies that have relied on a single source. Regarding the first data source, full-time employees from Canada and the US were recruited via Amazon Mechanical Turk to report their daily work experiences. Prior research has demonstrated the validity of such a sample [34]. In total, 157 individuals completed the online surveys powered by Qualtrics. The second source was the alumni base of a private US college in New York State. An invitation to participate was electronically sent to the alumni. Five hundred and forty people expressed interest, and of these, 336 completed the surveys (62.22%). We also analyzed the data controlling for sample type and present the results as part of our analysis.

Although the hypotheses herein are articulated at the individual level and not the day level (between- rather than within-person), we collected data during five consecutive workdays (Monday thru Friday). Some fluctuations in these perceptions could be attributed to factors associated with the day the person was asked to reflect back on their work stressors. For example, predictable changes in affect occur throughout the week as exemplified by clichés like ’Thank God It’s Friday’ [35]. Since these daily fluctuations are integral in how employees construct their perceptions of experienced stressors, their appraisals, and their perceived engagement resulting from these appraisals, our aggregate measure should include and not exclude those daily perceptual differences. Taking measures on five consecutive workdays will increase the validity of the between-person analysis as the aggregate measures for all participants would be based on the same combination of workdays. After removing six participants for failing all the attention questions, the final sample consisted of 487 full-time employees (ages from 21 to 74, *M* = 38.00, SD = 11.80), with 60.2% identifying as female and having an average tenure of 7.11 years (SD = 7.45).

#### 3.1.1 Ethics statement

Participants’ consent was procured electronically prior to commencing with the electronic questionnaire. Participants read the following statement "I have read and understood this form. I have had the chance to ask questions and any questions have been answered. I agree to participate in this research under the conditions described. I have carefully studied the above and understand this agreement. By pressing the button below, I freely consent and voluntarily agree to participate in this study." Then pressed a button that stated, "I understand and agree to participate," to indicate consent. Ethics approval was received by Concordia University for research involving human participants (# IRB 30009139 and from Ithaca College for research involving human participants (# IRB 0218-16bx1).

### 3.2 Measures

#### 3.2.1 Challenge and hindrance stressors

We used the 16-item measure by Rodell and Judge [34]. It assesses challenge stressors using eight items to measure workload, job responsibility, time urgency, and job complexity. A sample item includes, "Today, my job has required me to work very hard." It also measures hindrance stressors using eight items to tap red tape, role ambiguity, role conflict, and hassles. A sample item is, "Today, I have had to go through a lot of red tape to get my job done." This instrument employs a seven-point scale ranging from 1 (*strongly disagree*) to 7 (*strongly agree*). The averages of the daily Cronbach alphas for the challenge and hindrance stressor measures separately, were .83 and .86, respectively. The reliability of the entire 16-item measure was .87.

#### 3.2.2 Challenge and hindrance stress appraisals

Based on an existing manipulation [8, 36], Webster and colleagues [17] measured appraisals by presenting participants with clear definitions of hindrance and challenge stressors. A similar approach was used here. Specifically, following exposure to the concepts, participants evaluated each stressor item, regardless of whether it was a challenge or a hindrance stress item, and were then asked to evaluate on a scale from 1 (*strongly disagree*) to 7 (*strongly agree*) how much they "experienced this as a challenge" and how much "they experienced this as a hindrance." This renders each stressor to be left with the same number of challenge and hindrance appraisal items, a method that has been used in recent empirical work on stress appraisals [18]. As such, while stressors in the model were either challenge or hindrance stressors, the appraisal measures, per the transactional theory of stress and our rationale, were overall measures of the challenge and hindrance experienced due to all stressors. The rationale for such an approach resides in the notion that when evaluating a stressor, a respondent will be appraising the level of the stressor. Therefore, to capture the various appraisals, we need to assist the participant in focusing on certain aspects of the stressor. Asking about specific types of appraisals, after clearly defining them will help the respondent think about the challenging or hindering aspects of the stressor [34, 36]. Cronbach alpha estimations of challenge and hindrance appraisal measures were .92 and .93, respectively.

#### 3.2.3 Stress mindset

We used the Stress Mindset Measure-General [21], which includes eight items on a five-point scale, from 1 (*strongly disagree*) to 5 (*strongly agree*). A sample item is "Experiencing stress facilitates my learning and growth" (α = .92).

#### 3.2.4 Work engagement

We used the nine-item Utrecht Work Engagement Scale [37], a dominant engagement instrument in the field. A sample item is, "At my work today, I felt bursting with energy." All items were scored on a seven-point scale, from 1 (*strongly disagree*) to 7 (*strongly agree*) (α = .93). To reduce fatigue and, consistent with extant diary studies [38], a seven-item measure was used on Days 2 to 5 (α = .93).

## 4 Results

### 4.1 Aggregation analyses

We first verified whether data aggregation was warranted before testing the between-person hypotheses. To do so, we followed established procedures [39] that demanded we compute the interrater agreement index r_WG_(j) followed by the interrater reliability indices ICC(1) and ICC(2). We calculated the agreement index for each participant on every variable of interest. Before doing so, following the same best practices, we tried to select one or more defendable null distribution(s) that represent(s) a total lack of agreement. Since we did not have any a priori ideas about biases, we used the rectangular distribution coupled with a slightly skewed distribution. The latter represents a more conservative evaluation of the indices.

LeBreton and Senter [39] suggested a detailed set of standards when making a decision about whether or not the agreement index justifies aggregation with agreement indices below .30 indicating lack of agreement, those between .31 and .50 indicating weak agreement, between .51 and .70 indicating moderate agreement, between .71 and .90 indicating strong agreement, and above .90 indicating very strong agreement. The means and medians of the indices for all variables were larger than .70, with most falling above .90. Scrutinizing each variable individually [39], we found that the percentage of participants who had an agreement index above the traditional .70 cutoff point ranged between 88.11% and 98.19% for the uniform distribution and between 73.57% and 96.38% for the conservative skewed distribution. We also followed best practice recommendations by testing the extent to which the computed agreement values were significantly different from zero. The test was significant for the indices of all the variables (work engagement *F* ratio = 8.20, *p* = .00; stress mindset *F* ratio = 35.41, *p* = .00; challenge stressors *F* ratio = 9.31, *p* = .00; hindrance stressors *F* ratio = 14.01, *p* = .00; challenge appraisals *F* ratio = 13.76, *p* = .00; hindrance appraisals *F* ratio = 13.97, *p* = .00). All in all, the r_wg(j)_ agreement indices support aggregation. We then examined the ICC(1) and ICC(2) to determine if they also supported aggregation. While ICC(2) represents the reliability of group means, ICC(1) is the variance that can be attributed to group membership and can be looked at as an effect size estimate (i.e., the effect of group membership on the variance in an individual’s ratings). Researchers have thus suggested that ICC(1) ’s of .01, .10, and .25 are indicative of small, moderate, and large effects, respectively. Moreover, ICC(1) is important because one can only obtain large ICC values by having good interrater reliability and agreement [39, 40]. Although Likert-type formats tend to underestimate both ICC(1) and ICC(2), our ’variables’ ICC(1) values ranged between .37 (engagement) and .74 (stress mindset), and their ICC(2) values ranged between .88 (engagement) and .97 (stress mindset), showing a large effect of group membership and a reliable group mean. This evidence supports aggregation.

### 4.2 Hypothesis testing: Work engagement

Correlations, means, and standard deviations are presented in Table 1.

**Table 1:** Descriptive statistics and intercorrelations.

		**Mean**	**SD**	**1**	**2**	**3**	**4**	**5**	**6**	**7**	**8**	**9**	**10**	**11**	**12**
**1**	**Age**	**38.00**	**11.80**												
**2**	**Challenge Stressors**	**4.40**	**1.09**	**.14** **	**.86**										
**3**	**Challenge Appraisals for Challenge Stressors**	**4.32**	**1.09**	**.15** **	**.72** **	**.89**									
**4**	**Hindrance Appraisals for Challenge Stressors**	**2.87**	**.95**	**.04**	**.30** **	**.26** **	**.89**								
**5**	**Hindrance Stressors**	**2.98**	**1.04**	**.03**	**.50** **	**.31** **	**.57** **	**.81**							
**6**	**Challenge Appraisals for Hindrance Stressors**	**3.34**	**1.09**	**.17** **	**.42** **	**.70** **	**.53** **	**.48** **	**.88**						
**7**	**Hindrance Appraisals for Hindrance Stressors**	**3.29**	**1.18**	**.04**	**.39** **	**.45** **	**.76** **	**.69** **	**.59** **	**.90**					
**8**	**All Stressors**	**3.69**	**.92**	**.10** *	**.87** **	**.60** **	**.50** **	**.86** **	**.52** **	**.62** **	**.87**				
**9**	**Challenge Appraisals for All Stressors**	**3.83**	**1.01**	**.17** **	**.62** **	**.92** **	**.43** **	**.43** **	**.92** **	**.56** **	**.61** **	**.92**			
**10**	**Hindrance Appraisals for All Stressors**	**3.08**	**.10**	**.05**	**.37** **	**.39** **	**.92** **	**.68** **	**.60** **	**.95** **	**.60** **	**.53** **	**.93**		
**11**	**Stress Mindset**	**3.30**	**1.24**	**-.06**	**.04**	**.19** **	**.00**	**.03**	**.26** **	**.05**	**.04**	**.24** **	**.03**	**.92**	
**12**	**Work Engagement**	**3.00**	**.83**	**.11** *	**.29** **	**.38** **	**-.27** **	**-.19** **	**.16** **	**-.16** **	**.07**	**.29** **	**-.22** **	**.28** **	**.93**

*Note*: N = 487;

** Correlation is significant at the .01 level,

* Correlation is significant at the .05 level,

^†^ Correlation is significant at the .10 level.

Reliabilities are presented in bold on the diagonal.

As noted, the stress-work engagement relationship is still unclear [11]. To shed some light on this relationship, we first looked at how well stressors predicted engagement in general. *Undifferentiated* stressors were unrelated to engagement (B = .06, *p* = .14, C.I. [-.020 .140]) with the model explaining only about .40% of the variance in engagement (R = .067). Results were radically different using *differentiated* stressors. Challenge stressors positively predicted engagement (B = .39, *p* = .00, C.I. [.322 .460]), while hindrance stressors negatively predicted it (B = -.35, *p* = .00, C.I. [-.425 -.281]), with the model explaining 23.3% of the variance in engagement (R = .483). Though Mazzola and Disselhorst’s [14] findings were similar in direction, they lacked significance. We then used the more proximal challenge and hindrance appraisals to predict engagement and found improved results in both effect sizes and variance explained. Challenge appraisals positively predicted engagement (B = .48, *p* = .00, C.I. [.402 .548]) while hindrance stressors negatively predicted engagement (B = -.44, *p* = .00, C.I. [-.516 -.367]), with the model explaining about 28.8% of the variance in engagement (R = .537).

Second, for hypotheses testing that included mediation and moderation, we used conditional process analysis (PROCESS version 2.16, Models 4 and 58) [41], which calculates all model paths simultaneously. The analysis of the engagement model includes four moderated relationships, and considering that the employed measures show high reliabilities, PROCESS is useful in this case as it alleviates issues with non-normal interaction terms by using bootstrapping via repeated sampling with replacement. Results produced by PROCESS, in the case of highly reliable variables, converge greatly with those produced using advanced SEM techniques [42] and avoid known issues associated with the modeling and analysis of latent variable interaction [43]. We used the recommended 10,000 bootstrap samples. We started the analyses with a purely mediational model (PROCESS Model 4) in which challenge and hindrance appraisals mediated the relationships between challenge and hindrance stressors and work engagement. The results for this model are presented in the upper parts of Table 2, with the various direct effects presented first, followed by the mediational analysis. PROCESS Model 58 was used to test the fully moderated model. Results for the moderation analyses are shown at the bottom of Table 2. The Table also identifies what effect size corresponds to what hypothesis is being tested.

**Table 2:** Full results for work engagement.

**Mediation Analysis (Direct Effects)**	**Effect (SE)**	***t***	***p***	**95% LLCI**	**95% HLCI**
**Outcome: Challenge Stress Appraisal**					
***H1***- Challenge Stressors	.50(.04)	13.25	.00	.424	.572
***H3***- Hindrance Stressors	.16(.04)	3.98	.00	.079	.234
**Outcome: Hindrance Stress Appraisal**					
***H2***- Challenge Stressors	.04(.04)	1.09	.27	-.031	.108
***H4***- Hindrance Stressors	.63(.04)	17.18	.00	.560	.704
**Outcome: Work Engagement**					
***H9***- Challenge Stress Appraisal	.35(.04)	8.20	.00	.266	.433
***H10***- Hindrance Stress Appraisal	-.32(.05)	-7.05	.00	-.410	-.231
Challenge Stress	.23(.04)	5.98	.00	.154	.305
Hindrance Stress	-.21(.04)	-4.75	.00	-.290	-.120
**Mediation Analyses: Work Engagement**	**Effect (BootSE)**			**Boot LLCI**	**Boot HLCI**
***H13***- Challenge Stressors→Challenge Appraisal→Work Engagement	.17(.02)			.127	.230
***H16***- Challenge Stressors→Hindrance Appraisal→Work Engagement	-.01(.01)			-.040	.011
***H14***- Hindrance Stressors→Challenge Appraisal→Work Engagement	.05(.02)			.020	.095
***H15***- Hindrance Stressors→Hindrance Appraisal→Work Engagement	-.20(.03)			-.268	-.141
**Moderation Analyses**	**Effect (SE)**	***t***	***p***	**95% LLCI**	**95% HLCI**
**Outcome: Challenge Stress Appraisal**					
***H5***- Challenge Stressors × Mindset Interaction	.02(.03)	.65	.52	-.033	.066
***H7***- Hindrance Stressors × Mindset Interaction	.07(.03)	2.79	.01	.021	.123
**Outcome: Hindrance Stress Appraisal**					
***H6***- Challenge Stressors × Mindset Interaction	-.06(.02)	-2.38	.02	-.107	-.010
***H8***- Hindrance Stressors × Mindset Interaction	-.10(.03)	-4.17	.00	-.153	-.055
**Outcome: Work Engagement**					
***H11***- Challenge Stressor Appraisal × Mindset Interaction	-.04(.03)	-1.58	.11	-.091	.010
***H12***- Hindrance Stressor Appraisal × Mindset Interaction	.08(.03)	2.89	.00	.026	.136

*Note*: *N* = 487; SE = standard error, LLCI = lower-level confidence interval, HLCI = higher-level confidence interval.

Challenge appraisals were positively related to both challenge stressors (Effect = .50, *p* = .00, C.I. [.424 .572]; controlling for data source: Effect = .50, p = .00, C.I. [.429 .574]) and hindrance stressors (Effect = .16, *p* = .00, C.I. [.079 .234]; controlling for data source: Effect = .11, p = .00, C.I. [.035 .191]). Hindrance appraisals were positively related to hindrance stressors (Effect = .63, *p* = .00, C.I. [.560 .704]; controlling for data source: Effect = .59, p = .00, C.I. [.518 .664]) but not to challenge stressors (Effect = .04, *p* = .27, C.I. [-.031 .108]; controlling for data source: Effect = .04, p = .23, C.I. [-.026 .110]). As such, support was found for Hypotheses 1, 3, and 4, but not for 2. Overall, challenge appraisals positively predicted work engagement (Effect = .35, *p* = .00, C.I. [.266 .433]; controlling for data source: Effect = .35, p = .00, C.I. [.269 .438]) and hindrance appraisals negatively predicted engagement (Effect = -.32, *p* = .00, C.I. [-.410 -.231]; controlling for data source: Effect = -.32, p = .00, C.I. [-.406 -.226]), thus supporting Hypotheses 9 and 10, respectively.

Third, PROCESS includes both independent variables in the analysis but only dubs one as the independent variable, with the other entered as a covariate. To get the effect sizes for the second independent variable, the user reruns the analysis twice [41], once with challenge stressors as the independent variable and hindrance stressors as a covariate (and the reverse assignment in another iteration) [44: p.154]. Since both models are statistically identical, all model parameters remain the same; the two analyses provided the needed various indirect effects. While hindrance appraisals did not mediate the link between challenge stressors and engagement (Effect = -.01, Boot SE = .01 C.I. [-.040 .011]; controlling for data source: Effect = -.01, Boot SE = .01 C.I. [-.041 .010]), challenge appraisals did (Effect = .17, Boot SE = .03 C.I. [.127 .230]; controlling for data source: Effect = .18, Boot SE = .03 C.I. [.129 .235]), thereby failing to support Hypothesis 16 but confirming Hypothesis 13. When assessing the indirect effect of hindrance stressors on engagement, both mediations through challenge appraisals (Effect = .05, Boot SE = .02 C.I. [.020 .095]; controlling for data source: Effect = .04, Boot SE = .02 C.I. [.005 .080]) and hindrance appraisals (Effect = -.20, Boot SE = .03 C.I. [-.268 -.141]; controlling for data source: Effect = -.19, Boot SE = .00 C.I. [-.248 -.130]) were significant supporting Hypotheses 14 and 15, respectively.

**Fig 2:**
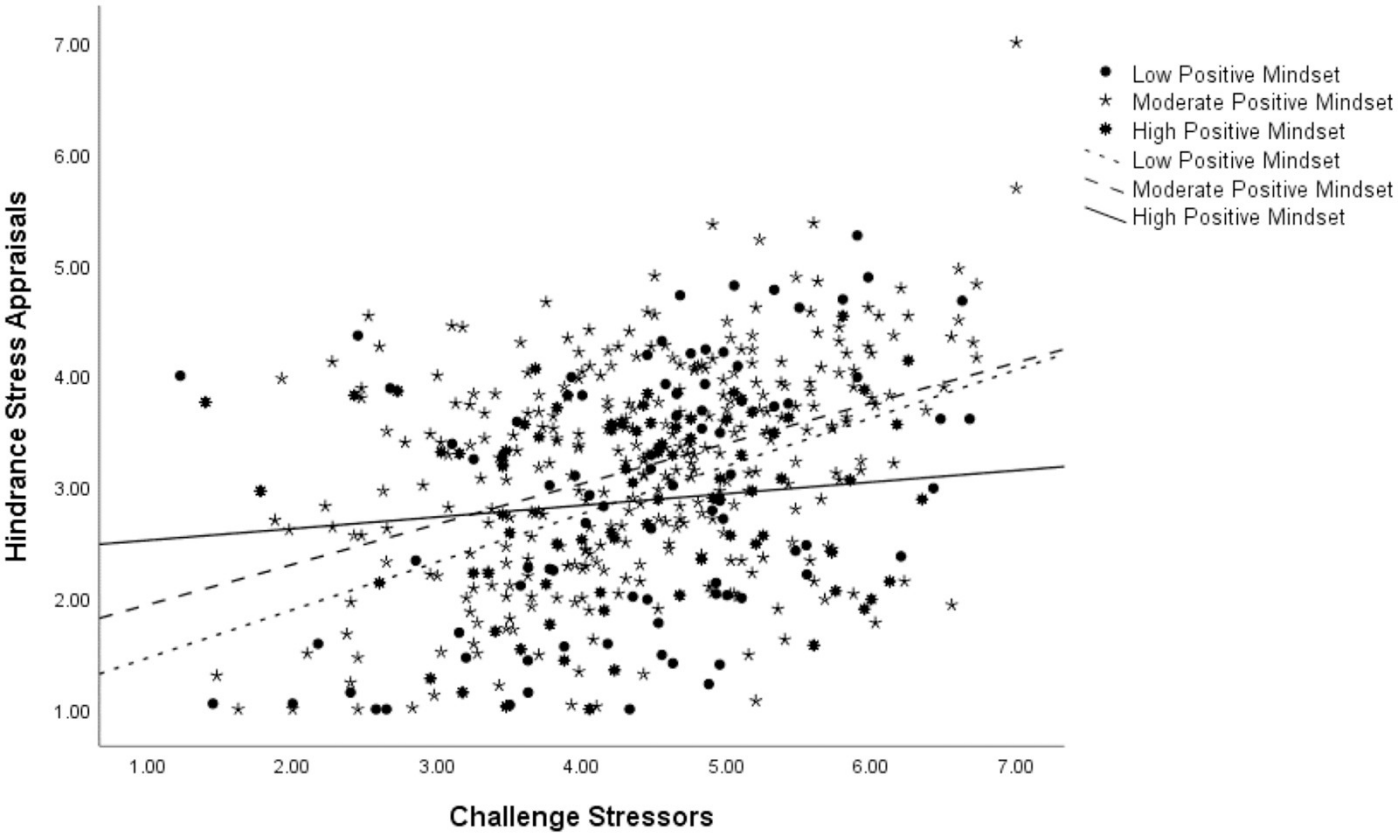
Moderation analysis for the challenge stressor-hindrance appraisal relationship at three levels of stress mindset.

PROCESS Model 58 is a mediational model with one variable moderating both the independent variable-to-mediator link and one connecting the mediator to the dependent variable. Stress mindset did not moderate the relationship between challenge stressors and challenge appraisals (Effect = .02, *p* = .52, C.I. [-.033 .066]; controlling for data source: Effect = .03, p = .29, C.I. [-.023 .076]), failing to support Hypothesis 5. It did, however, moderate the challenge stressor-to-hindrance appraisal link (Effect = -.06, *p* = .02, C.I. [-.107 -.010]; controlling for data source: Effect = -.05, p = .05, C.I. [-.095 .000]) supporting Hypothesis 6. Fig 2 depicts the associations between challenge stressors and hindrance appraisals at three levels of stress mindset: low (1 SD below the mean), high (1 SD above the mean), and moderate representing in-between levels. We should mention that controlling for the source of the data, the interaction was still significant, but the significance level and confidence intervals were borderline.

Simple-slope analyses revealed that individuals with a high positive mindset show the least positive slope (Effect = .11, *p* = .25, C.I. [-.074 .284]) followed by those with a moderate positive mindset (Effect = .36, *p* = .00, C.I. [.273 .453]) and, finally, those with a low positive stress mindset (Effect = .43, *p* = .00, C.I. [.228 .636]). Simply put, as the level of reported challenge stressors increased, all participants were reporting higher levels of hindrance appraisals but that trend was the weakest among those that had a highly positive stress mindset and strongest among their negative mindset counterparts. Stress mindset is thus reducing the perception of hindrance in challenge stressors.

**Fig 3:**
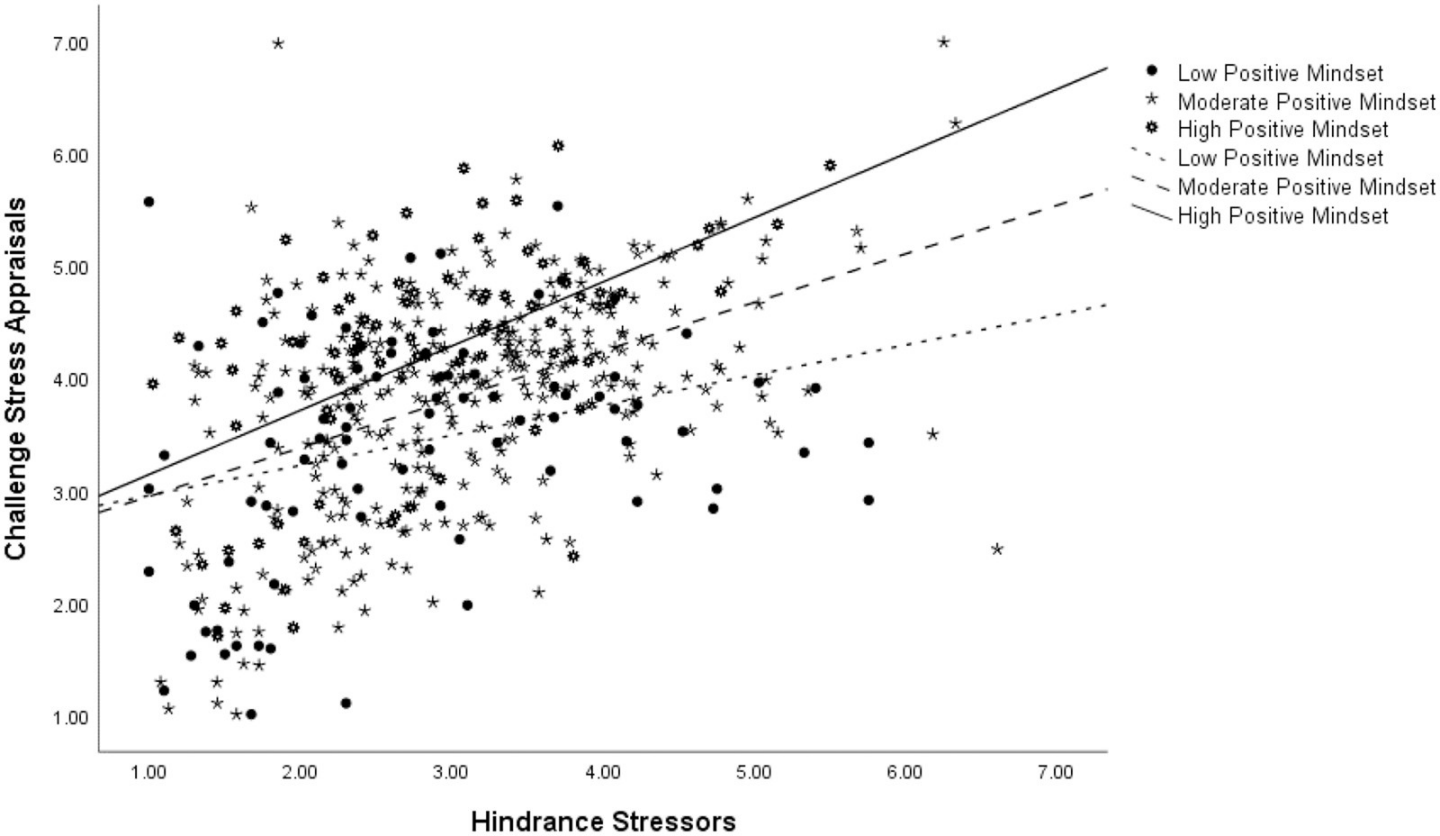
Moderation analysis for the hindrance stressor-challenge appraisal relationship at three levels of stress mindset.

Next, we looked at the moderating role of stress mindset on the hindrance stressor-to-appraisal relationship. We surmised that people having a more positive stress mindset will be more likely to realise the challenging aspects of hindrance stressors compared to those with a less positive stress mindset. Mindset, in fact, moderated the relationship between hindrance stressors and challenge appraisals (Effect = .07, *p* = .01, C.I. [.021 .123]) (Controlling for data source: Effect = .08, p = .00, C.I. [.027 .128]) supporting Hypothesis 7. This interaction was in the expected direction, as illustrated in Fig 3.

**Fig 4:**
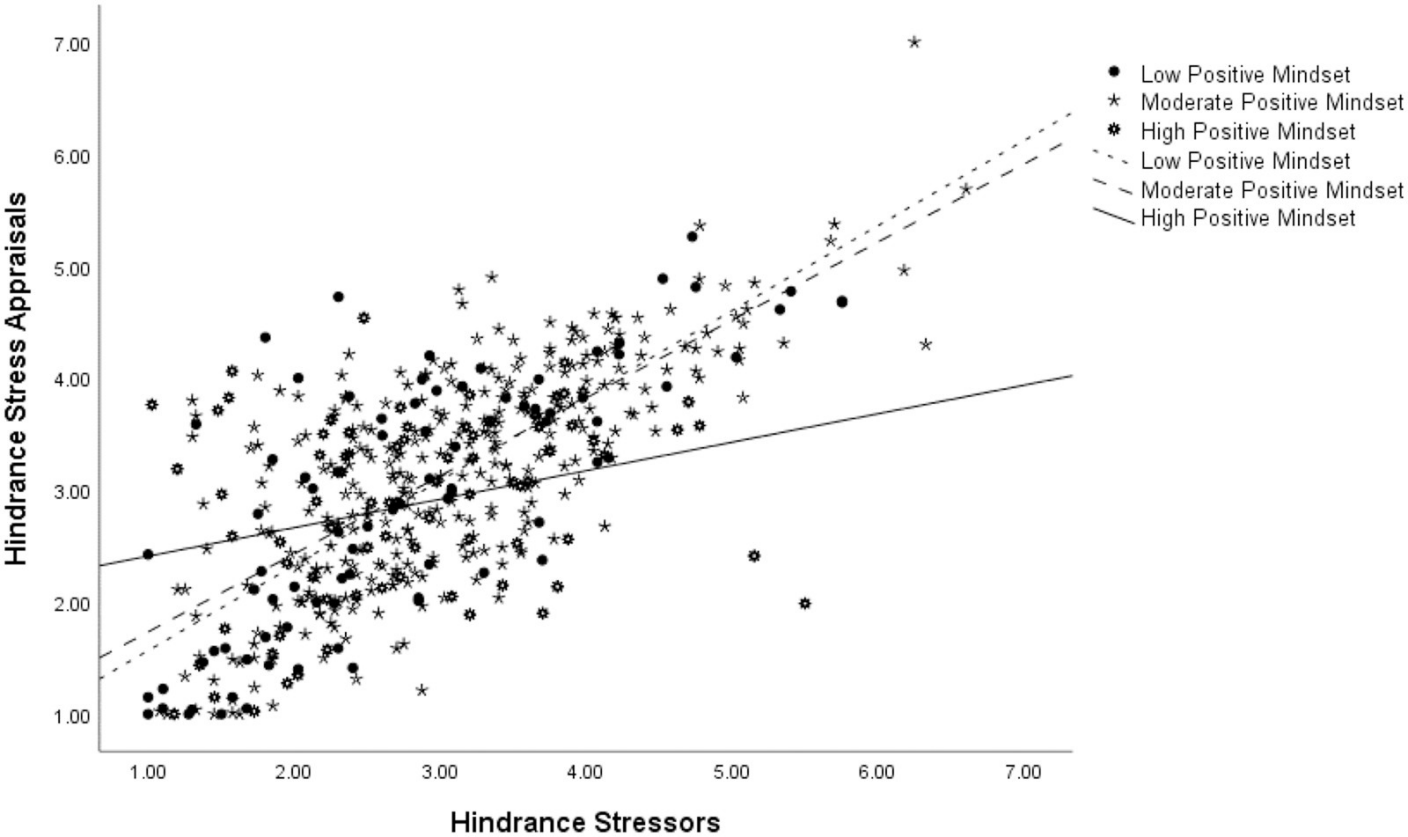
Moderation analysis for the hindrance stressor-hindrance appraisal relationship at three levels of stress mindset.

Simple-slopes analysis demonstrated that the effects for all three levels were significant, the largest being for individuals with a high positive stress mindset (Effect = .57, *p* = .00, C.I. [.375 .772]), followed by those with a moderate stress mindset (Effect = .43, *p* = .00, C.I. [.339 .527]), and finally by those with the lowest stress mindset (Effect = .27, *p* = .00, C.I. [.087 .450]). Thus, as the level of reported hindrance stressors increased, all participants were reporting higher levels of challenge appraisals but that trend was the strongest among those that had a highly positive stress mindset and weakest among their negative mindset counterparts. Stress mindset is thus allowing individuals to see the challenging aspects of hindrance stressors. Finally, we expected a high positive stress mindset to buffer the association between hindrance stressors and hindrance appraisals. The moderation was significant and in the predicted direction (Effect = -.10, *p* = .00, C.I. [-.153 -.055]; controlling for data source: Effect = -.10, p = .00, C.I. [-.147 -.050]) supporting Hypothesis 8 (Fig 4).

Again, a simple-slopes analysis revealed that the effects for all three levels were significant, with the effect being the weakest for individuals reporting high positive stress mindset (Effect = .25, *p* = .01, C.I. [.071 .438]), followed by an effect slightly stronger for those with a moderate mindset (Effect = .70, *p* = .00, C.I. [.622 .770]), and finally the strongest effect for those reporting the lowest levels of stress mindset (Effect = .76, *p* = .00, C.I. [.619 .898]). As one can see, as the level of reported hindrance stressors increased, all participants were reporting higher levels of hindrance appraisals, but that trend was the weakest among those that had a highly positive stress mindset and strongest among their negative mindset counterparts. Stress mindset is again reducing the perception of hindrance in hindrance stressors.

**Fig 5:**
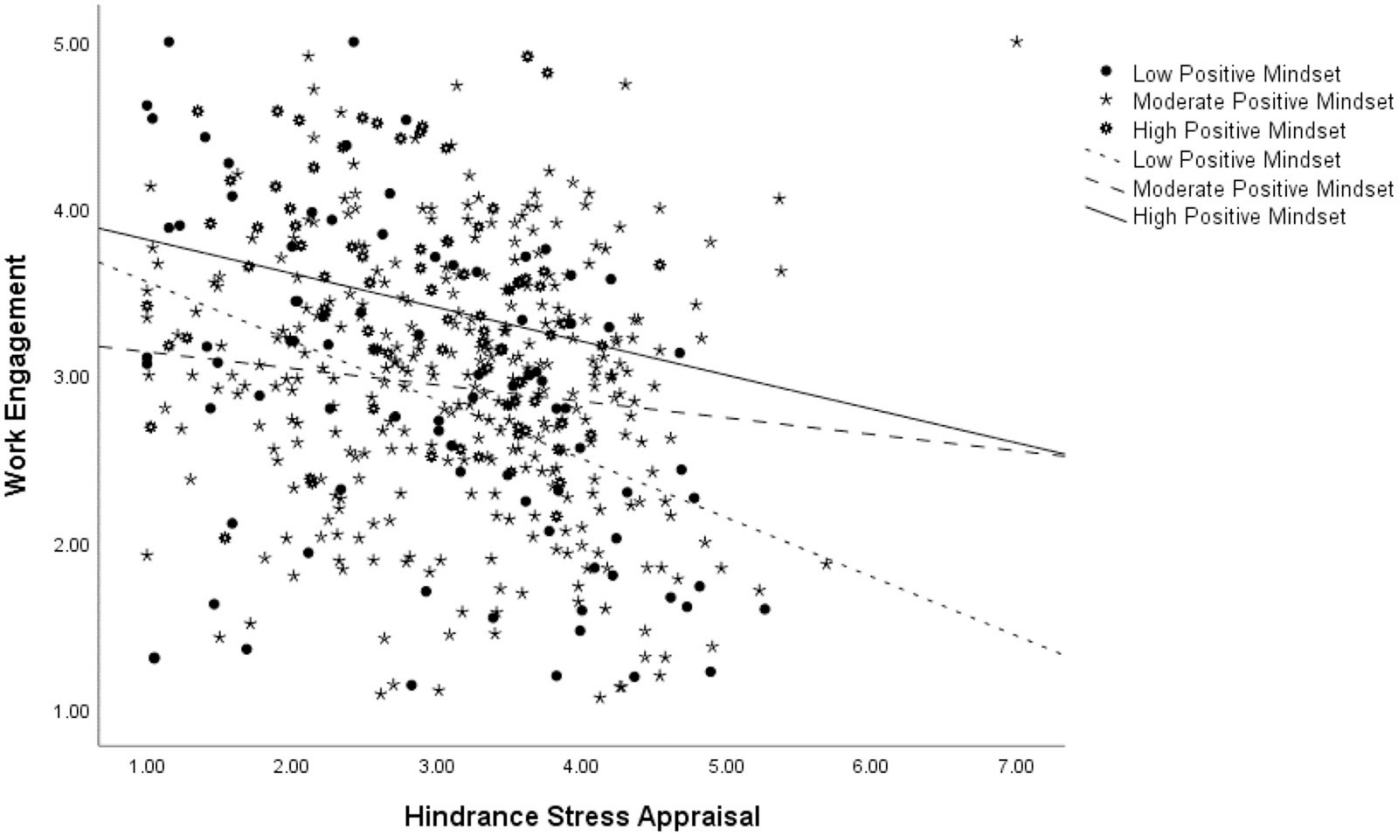
Moderation analysis for the hindrance appraisal-work engagement relationship at three levels of stress mindset.

Significant positive and negative effects were found for challenge and hindrance appraisals on engagement, respectively. However, stress mindset did not moderate the association between challenge appraisals and work engagement (Effect = -.04, *p* = .11, C.I. [-.092 .009]; controlling for data source: Effect = -.05, p = .07, C.I. [-.100 .004]), failing to support Hypothesis 11. Controlling for the data source, this interaction became marginally significant. We also predicted that a higher positive stress mindset would buffer the negative effects of hindrance appraisals on engagement. Indeed, a positive stress mindset was found to play a significant moderating role between hindrance appraisals and engagement (Effect = .08, *p* = .00, C.I. [.026 .136]; controlling for data source: Effect = .08, p = .00, C.I. [.029 .140]). Fig 5 shows this interaction at the three levels of mindset.

When stressors were appraised as hindering, simple-slope analyses show that the sharpest significant decrease in engagement occurred for individuals with the lowest positive stress mindset (Effect = -.35, *p* = .00, C.I. [-.520 -.187]). A smaller decrease in engagement was observed for individuals with moderate (Effect = -.10, *p* = .03, C.I. [-.187 -.011]) and high positive stress mindset (Effect = -.20, *p* = .03, C.I. [-.384 -.023]). Moreover, individuals with a high positive stress mindset reported higher levels of engagement at *every* level of appraised hindrance compared to individuals with a lower positive stress mindset. In sum, these findings provide strong support for Hypothesis 12.

## 5 Discussion and implications for managerial praxis

### 5.1 Discussion

This paper addressed two neglected issues in prior research. First, it employed a measure of appraisals that concurs with the precise definitions of challenge and hindrance stress according to the transactional theory of stress [9]. Second, it gathered longitudinal data about a typical five-day work week from full-time employees about prevalent chronic work stressors rather than idiosyncratic acute ones, thus capturing employees’ emergent perceptual evaluations of the various variables accounting for the various fluctuations in these evaluations over the week. Our findings support Lazarus’s [9] original assumption, namely that individuals appraise stressors, irrespective of how researchers elect to categorise them, as both challenging and hindering. Moreover, they refute the longstanding one that challenge and hindrance stressors are appraised solely as challenges and hindrances, respectively. If one inspects the correlations between challenge and hindrance stressors and each of their respective appraisals, one finds a strong relationship between each stressor category and the appraisal fitting that category. One reason why using dichotomised stressors is effective in research has to do with measurement convenience. However, this strategy neglects other relationships (e.g., challenge stressors to hindrance appraisals; hindrance stressors to challenge appraisals), which may threaten internal validity through the development of unintended hypotheses that violate theories on which they are based and, consequently, lead researchers to erroneous findings.

Specifically, this paper contributes by offering evidence to support the inclusion of both types of appraisals in any testable model that tries to explain a workplace outcome associated with a particular stressor. A key takeaway is that any stressor could be appraised as both challenging and hindering. Also, the work adds to existing knowledge by the inclusion of stress mindset as a boundary condition to how stressors are appraised. We predicted that individuals with a more positive stress mindset tend to see more opportunities or fewer obstacles in both challenge and hindrance stressors compared to their counterparts having a less positive mindset. For the most part, these predictions were supported, and mindset was found to play a critical role in how people appraise the environmental stressors they encounter.

We accurately predicted that greater challenge appraisals would be associated with greater reported work engagement, with the opposite occurring for hindrance appraisals. These palpable positive and negative relationships, respectively, were not seen when undifferentiated stressors were used to predict engagement. Also shown was how including appraisals with differentiated stressors improved the variance explained in engagement compared to a model housing differentiated stressors only. Although differentiated challenge and hindrance stressors foretold engagement positively and negatively, respectively, this could have been due to high correlations between stressors and their matched appraisals and does not imply that the associations between stressors and their unmatched appraisals should be ignored. With the analyses presented here, we hope that researchers will understand this germane point as it is one of the foundational principles of most stress theories. In addition, appraisals constituted a paramount mechanism by which stressors influenced engagement. These findings show that including appraisals can align the challenge-hindrance stress model with the theory on which it was developed.

Last, stress mindset was instrumental not only in how appraisals formed but also in how they translated to outcomes, namely work engagement. Although mindset did not moderate the challenge appraisal-to-engagement relationship, one explanation for this could be that these appraisals are void of threats and, as such, one’s beliefs about stress matter less in these outcomes. In short, when one evaluates a group of stressors positively, stress mindset might not be influential in how that evaluation influences engagement. That was not the case with hindrance appraisals for which mindset played a buffering effect. In this case, and since the evaluation is that the stressors are hindering, believing that stress is debilitating will exacerbate the effects of that appraisal. A higher positive mindset, on the other hand, significantly attenuated the negative effects of hindrance appraisals on engagement.

### 5.2 Implications for managerial praxis

When it comes to managerial practice, it is critical for managers not to assume that a stressor falling into one category (challenge, hindrance) will be appraised by all employees commensurately. Doing so would mean casting aside the role of individual differences, the importance of the intensity of stressor dimensions (high, moderate, low), and people’s range of interpretations of stressful stimuli. For instance, workload, typically categorised as a challenge stressor, may be appraised as challenging *and* hindering depending on how strenuous it is. During the ongoing COVID-19 pandemic, intensive care unit nurses work 18-hour shifts and experience a workload that is different from most other occupations. The same could be said about a hindrance such as role conflict, defined as incompatible expectations needed for a job. Some degree of role conflict, one could argue, helps spur motivation in workers who appraise it as a challenge because it requires them to use a plethora of skills.

That being said, moderate or high levels of role conflict, for example, will likely lead one to burnout. Managers, therefore, need to be aware of their ’members’ skillset and to understand what job features each member evaluates as growth-inducing and enjoyable as well as goal-thwarting and frustrating, but also be mindful that even this approach is not a silver bullet to improve work-related outcomes as other factors can interact to influence such appraisals. Being able to notice the hindering and challenging qualities of a job, even if that job is deemed to have challenging or hindering characteristics, may also be a step in building trust among employees who will feel valued because their interests are considered.

The second implication is that managers must understand that, regardless of a job’s stressors, any stressors appraised as challenges will be positively related to engagement, and those appraised as hindrances will be negatively related to it. While this may be intuitive, the fact remains that most empirical work has not considered the interpretative nature of stressors and the more proximal role that appraisals play in predicting work outcomes such as engagement. Together, these findings support the tenets of goal-setting theory [45], which advocates that goals become motivational whenever they are challenging. As such, managers ought to rethink job design to draw out the most challenging aspects of a given job.

Third, building on the momentum of stress mindset research [46], another consideration is based on the idea of making employees see the good in stress. The narrative surrounding how people in our society think about stress is that it is pernicious and must be mitigated. Individuals having a positive stress mindset who choose to see the benefits of stress report stressors as being less (more) hindering (challenging) in their work. Thus, having a positive stress mindset is not the result of environmental context (e.g., resource abundance) but rather a more or less stable cognitive individual difference that could be cultivated and shaped through practice [47]. The good news, as noted, is that stressors thought of as hindrances are not appraised as such by all individuals. Another key finding is that individuals high in positive stress mindset not only appraised hindrance stressors as more challenging but also appraised both challenge and hindrance stressors as less hindering. In other words, those having a positive stress mindset see more challenge in the debilitating aspects of work and less hindrance, whether or not the work is enhancing or debilitating. Which manager would not wish to have such members be a part of the team? As scholars, we need to be cautious of potential drawbacks to having what seems to be a panacea in positive stress mindset. To the extent that the latter does not encourage a worker to take on more stress than needed, there is no empirical proof suggesting the pitfalls of having such a mindset. So, how does one change one’s stress mindset?

One of the first interventions carried out by Crum and her colleagues took place at a global company in the financial sector [21]. Organisational interventions of this sort prime workers to use stress as a means to boost resilience (physical and psychological), sharpen one’s focus, and cultivate interpersonal relationships. While not radically altering one’s perception of stress overnight, such exposure educates employees to begin endorsing a balanced view of stress in which they can appreciate its benefits. The key to priming is the cascade of behavioural changes that linger long after the intervention is complete; individuals subjected to brief interventions communicating how stress can benefit one’s life at work start taking on a more proactive stance when anticipating stressors. As they gradually accept that stress is inevitable, they are more likely than their negative stress mindset counterparts to plan how they will deal with the sources of stress, including seeking information and mentors. Employees who once had a negative stress mindset stopped avoiding or denying stress and instead began building their personal resources to tackle it head-on.

Another approach is to gravitate away from experimental designs where employees are randomly assigned to manipulated treatments and to conduct "open-label mindset interventions" [47: p.29]. Employees join a stress-management training program that explains the beneficial and detrimental aspects of stress and introduces them to the concept of stress mindset. They are explicitly told that the program will help them adopt and foster a more positive stress mindset by asking them to ponder on their unique positive and negative experiences with stress. Employees transform their stress by 1) noticing how it affects them, 2) accepting that it signals something one cares deeply about, and 3) using the energy it generates more productively instead of ruminating about it and/or engaging in aimless pursuits to avoid it.

Finally, practitioners must think about how they can create organisational cultures to engage their workforce for the long term rather than focus on the present. More than ever before, this is imperative because of two watershed events that have shaken organisational life globally in the last decade. The first is the financial crisis of 2008, and the second is the ongoing COVID-19 crisis. While the former is often framed as an economic crisis and the latter as a public health crisis, both events have adversely affected organisational cultures and work engagement across the globe more profoundly than any other environmental factor [1, 48]. Experts believe that these tipping points, however, also provide opportunities for organisations to recalibrate their efforts by focusing more on creating cultures that foster employee engagement. One way in which this could materialise is to evaluate the affective connections that workers have with their environments. In other words, the ’workers’ collective feelings toward the job should be good enough such that they will want to enact the behaviours needed to stay committed to, and to accomplish their goals within their organisations. Bowles and Cooper [48] advise that building a high-engagement work culture requires one to have a strong sense of identity and to draw upon the history and values of the organisation, its leadership and management, and its employees.

### 5.3 Limitations and avenues for future research

As with all works, this one is without its limitations. Employees reported their work experiences only at the *end* of each day, which might not reflect one’s true experiences *throughout* the day. This would be resolved by having a repeated-measures design, i.e., multiple data points over the course of a day. Such a strategy would have been more effective in garnering more data but might have led to undesirable issues (high dropout rate, respondent fatigue, and stress unrelated to work). For example, two hindrance stressors are hassles and role overload. Answering questions about such stressors, appraising them, and then evaluating work engagement three times per day might artificially inflate both stressors. One may increase the daily sampling instances by reducing dimensions, although this would threaten content validity for both challenge and hindrance stressors. Second, we used self-report measures, which were once criticized in stress research [49]. This, however, should not be problematic because our investigation involves perceptions that span behavioural, cognitive, and emotional evaluations of one’s subjective experiences at work. Lastly, eight stressor dimensions were used based on previous conceptualisations of challenge and hindrance stressors [8]. Though the list includes common stressors across occupations, it is by no means exhaustive. For example, physical demands, dangerous work conditions, and specific work reengineering due to COVID-19 are not included. Researchers must therefore acknowledge context prior to data collection. A final point about measurement is that we assumed and analyzed our variables as emergent constructs that encompass fluctuations in how they are perceived over the course of a week. Our rationale was to make sure that differences in daily experiences, critical to how people evaluate their work experiences, are included. However, researchers might be interested in looking at the cascading effects on a daily basis. Anecdotal and empirical evidence shows predictable variations in attitudes throughout the week. Studying these variations may lead to better managerial interventions to attenuate their adverse effects and boost their positive ones.

There is ample evidence surrounding the importance of stress mindset in both how appraisals are formed and how they affect work-related outcomes. However, stress mindset could be related to a myriad of constructs. Casper et al. [50] studied the effects of stress mindset on the relationship between workload (here, a challenge stressor) and its anticipation on coping efforts and found that individuals with a more positive stress mindset made more approach-coping efforts when they expected a higher workload. This could be a fruitful research area as coping includes behavioural and cognitive attempts to manage stress [51]. Coping styles could thus be influenced by one’s perceptions of the effects of stress and, in turn, influence work outcomes. Another avenue worth exploring is to include personality traits related to stress. For example, hardiness is associated with more commitment during stress situations, more perceived control over these situations, and a more positive perspective when faced with them [52].

Scholars could also scrutinise the magnitude of the stressor and its appraisal. Edwards et al. [53] found that the stressor’s magnitude influenced not only its evaluation as either hindering or challenging but also its influence on performance. This is consistent with our reasoning, which contends that challenge stressors are positively related to hindrance appraisals. Like hindrance stressors, challenge stressors draw energy and are associated with increased strain. Therefore, future research could explore at which appraisal level challenge stressors lose their positive impact on outcomes of interest. Time pressure has been found to be motivating and engagement-inducing when experienced for the short-term but hindered engagement after long-term exposure [54]. This is a worthwhile pursuit because increasing challenge stressors blindly might be detrimental to individuals with a negative stress mindset or to those lacking a problem-focused coping style.

Another area that is worth pursuing is integrating other boundary conditions in the study of the influence of stress mindset on how stressors are appraised and reacted to. In this study, we have shown that the way you perceive stress is of great importance in how you appraise work-related stressors and the effects of those appraisals on your work engagement. However, research has established that personality characteristics not only interact with various stressors to influence work-related outcomes (e.g., workload moderating the effects of perfectionism on workaholism [55]) but also influence how perceptions of stress translate into responses [56]. As such, integrating both personality and mindset could provide researchers with a clearer understanding of the stressors-to-response process.

Finally, another research avenue deals with considering added types of stressor appraisal categories. While hindrances impede one’s growth, threats are malignant demands that harm the individual [9]. For instance, the COVID-19 crisis has contributed to employee musings about the likelihood of getting sick, resulting in burnout. Such a stressor would not be considered a hindrance when one fears the worst for merely showing up to work. Therefore, future research would benefit greatly by exploring how the collective body of appraisals interacts with stress mindset to produce important workplace outcomes.
